# Prevention of Central Line-Associated Bloodstream Infections Through Educational Interventions in Adult Intensive Care Units: A Systematic Review

**DOI:** 10.7759/cureus.17293

**Published:** 2021-08-18

**Authors:** Maria Foka, Eleni Nicolaou, Theodoros Kyprianou, Lakis Palazis, Maria Kyranou, Elizabeth Papathanassoglou, Ekaterini Lambrinou

**Affiliations:** 1 Internal Medicine, Nicosia General Hospital, Nicosia, CYP; 2 Faculty of Nursing, Cyprus University of Technology, Limassol, CYP; 3 Faculty of Nursing, University of Alberta, Edmonton, CAN

**Keywords:** central venous catheters, infection, central line associated bloodstream infections, intensive care unit, education, intervention

## Abstract

Central line-associated bloodstream infections (CLABSIs) represent a severe systemic threat to patients admitted in ICUs and contribute to increased mortality, prolonged length of stay in ICUs, and increased costs. The majority of CLABSIs are preventable. The current systematic review aimed to investigate the effectiveness of educational methods on CLABSI rates in adult ICUs.

A systematic literature search was conducted using the electronic databases of Medline, Cumulative Index to Nursing and Allied Health Literature (CINAHL) Plus, and Cochrane Database of Systematic Reviews for studies published from the beginning of 1995 to March 2020. The terms used for the search were as follows: central venous catheters, infection, central line-associated bloodstream infections, intensive care unit, and education intervention in all possible combinations and using the word ‘and’ between them. Data were extracted independently and crosschecked by two authors using a standard data collection form. The quality of the studies included in the review was assessed using the Methodological Index for Non-randomized Studies (MINORS).

The current systematic review included 27 interventional studies of central line insertion or maintenance or both in adult ICU settings with documentation of the CLABSI incidence expressed per 1,000 catheter days. A large deviation between the length of time and type of educational interventions was found.

Statistical significance was found in all studies (except one) in terms of CLABSI reduction despite the large variation of the length or the type of the educational intervention. Continuing education on infection prevention may be necessary to maintain the post-intervention results and improve clinical outcomes.

## Introduction and background

Central venous catheters (CVCs) are the most important devices used in ICU patients, and they enable the administration of medications, ﬂuids, and blood products directly to the central venous system as well as hemodialysis therapy and hemodynamic monitoring [1]. Although they are extremely necessary tools, CVCs can expose critically ill patients to the risk of central line-associated bloodstream infections (CLABSIs). CLABSIs are deﬁned as bloodstream infections with an onset of at least 48 hours after the insertion of a central catheter, which is not related to another site [2]. These infections are associated with increased mortality and morbidity, increased length of stay, and increased hospitalization cost [3-7].

Studies have shown that simple interventions such as hand hygiene, maximal sterile barriers during catheter insertion, chlorhexidine skin disinfection, optimal catheter site selection, and daily review of line necessity with prompt removal of unnecessary lines can decrease the risk of CLABSIs [1,8-10]. Nevertheless, the International Nosocomial Infection Control Consortium (INICC) has stated that the pooled incidence of CLABSIs in INICC ICUs (ie, in Africa, Asia, Europe, and Latin America) is 4.9 infections per 1,000 central-line days, and it is nearly ﬁve times higher than those reported in the USA [11].

The latest data shows that regardless of the availability of evidence-based interventions summarized in the guidelines [12,13], CLABSI rates remain very high (2.7 per 1,000 catheter days) [14]. Bion et al. [9] concluded that the implementation of central-line insertion and maintenance bundles, which were first implemented by Pronovost et al. [8], signiﬁcantly reduced CLABSI incidence in ICUs. Despite the availability of evidence-based interventions and recommendations [15], the implementation of prevention strategies is usually insufficient due to reasons associated with low staff awareness, poor understanding of or disagreement with existing knowledge, failure to change institutional practice, and the lack of resources [16,17].

Several educational interventions such as lectures, seminars, simulations have been organized with the aim to reduce CLABSI rates [1]. However, their effectiveness has not been fully assessed. The present systematic review examines the impact of educational interventions on CLABSI rates in adult ICUs. The association between effectiveness and several characteristics of educational programs is also discussed. We relied on the hypothesis that educational interventions will have a positive effect on the prevention of CLABSIs.

## Review

Search strategy

A comprehensive literature review of Medline, Cumulative Index to Nursing and Allied Health Literature (CINAHL) Plus, and Cochrane Database of Systematic Reviews was conducted for studies published from the beginning of 1995 to March 2020. The search was conducted during February and March 2020.

Specifically, Medline was systematically searched through a combination of search terms: (((("Central Venous Catheters"[Mesh]) OR ("Central Venous Catheter"[Title/Abstract] OR CVC [Title/Abstract] OR "central line catheter"[Title/Abstract]))) AND (("Infection"[Mesh]) OR Infection[Title/Abstract]))) OR ("central line associated bloodstream infections" or clabsi))) AND (("Intensive Care Units"[Mesh]) OR (icu[Title/Abstract] OR "intensive care unit"[Title/Abstract] OR "critical care"[Title/Abstract])))) AND (("Education"[Mesh]) OR (education[Title/Abstract] OR learning[Title/Abstract] OR teaching[Title/Abstract])). Also CINAHL Plus with Full Text was searched (1995-March 2020) through a combination of search teams: ((MM "Central Venous Catheters" OR "Central Venous Catheter" OR CVC OR "central line catheter")) AND ((MM "Infection" OR Infection OR "central line associated bloodstream infections" or clabsi)) AND ((MM "Intensive Care Units" OR icu OR "intensive care unit" OR "critical care")) AND ((MM "Education" OR education OR learning OR teaching)). Cochrane Database of Systematic Reviews was systematically searched (1995-March 2020) through a combination of search teams: ("Central Venous Catheter" OR CVC OR "central line catheter") AND (Infection OR "central line associated bloodstream infections" or CLABSI) AND (icu OR "intensive care unit" OR "critical care") AND (education OR learning OR teaching). Extra studies were identified via reference lists and manually. Words used for the search were: central venous catheters, infection, central line associated bloodstream infections, intensive care unit, and education intervention in all possible combinations.

Study selection

Predefined selection criteria were set as follows.

Studies were included if:

· They reported educational interventions only for central venous lines (insertion or maintenance or both)

· They involved only adult ICU setting

· They documented CLABSI incidence expressed per 1,000 catheter days

· They made a comparison using a randomized or non-randomized study design, or an interrupted times series (ITS)

· They described an intervention (ie, lecture, simulation, seminar, workshop, feedback, bundle, checklist, etc.) to reduce CLABSI rates

Reviews, editorials, congress abstracts, or studies that did not report CLABSI incidence were excluded. We used studies in the English language only.

Inclusion criteria

We included all randomized controlled trials, studies that provided details on before and after the implementation of infection prevention control, as well as interrupted time-series analyses. Studies that examined the effectiveness of an educational intervention targeted at healthcare personnel for CLABSI prevention were selected. The primary outcome was the incidence of CLABSI. Studies that did not report the incidence of CLABSI as an outcome were excluded.

Data extraction

Data were extracted independently and crosschecked by authors using a standard data collection form. Author names, year of publication, sample size, settings, design, duration of the study, description of the intervention, the number of infections, and CVC days were among the extracted data. In case of discrepancies, a consensus was reached by discussion.

Quality assessment

We assessed the methodological quality of every trial for the risk of bias using the Methodological Index for Non-randomized Studies (MINORS) [18]. It consists of 12 questions (items) that evaluate the methodological and scientific value of published articles. Eight questions were selected for methodological assessment for non-randomized studies (A clearly stated aim, Inclusion of consecutive patients, Prospective collection of data, Endpoints appropriate to the aim of the study, Unbiased assessment of the study endpoint, Follow-up period appropriate to the aim of the study, Loss to follow-up less than 5%, Prospective calculation of the study size) and four additional questions in the case of comparative studies (An adequate control group, Contemporary groups, Baseline equivalence of groups, Adequate statistical analyses).

The items were scored as follows: 0 (not reported), 1 (reported but inadequate), or 2 (reported and adequate). The global ideal score was set as 16 for non-comparative (non-randomized studies) and 24 for comparative studies. All studies selected (27 studies) were non-comparative (non-randomized studies) with a top score of 16 points (Table 1) [5,19-44].

**Table 1:** Studies and MINOR scores MINORS: Methodological Index for Non-randomized Studies

Study	MINORS score
Mazi et al. (2014)	6/16
Guerin et al. (2010)	7/16
Ong et al. (2011)	7/16
Walz et al. (2015)	8/16
Perez Parra et al. (2010)	8/16
Azim et al. (2019)	8/16
Santana et al. (2008)	8/16
Galpern et al. (2008)	8/16
Lobo et al. (2010)	9/16
Barsuk et al. (2009)	9/16
Ilan et al. (2012)	9/16
Burden et al. (2012)	9/16
Render et al. (2011)	9/16
Paquet et al. (2019)	9/16
Leblebicioglu et al. (2013)	9/16
Exline et al. (2013)	10/16
Rosenthal et al. (2010)	10/16
Marra et al. (2010)	10/16
Alkhawaja et al. (2019)	10/16
Coopersmith et al. (2004)	10/16
Rosenthal et al. (2018)	10/16
Warren et al. (2004)	10/16
Park et al. (2017)	11/16
Hansen et al. (2014)	12/16
Khalid et al. (2013)	12/16
Jaggi et al. (2013)	12/16
Allen et al. (2014)	13/16

Results

**Figure 1:**
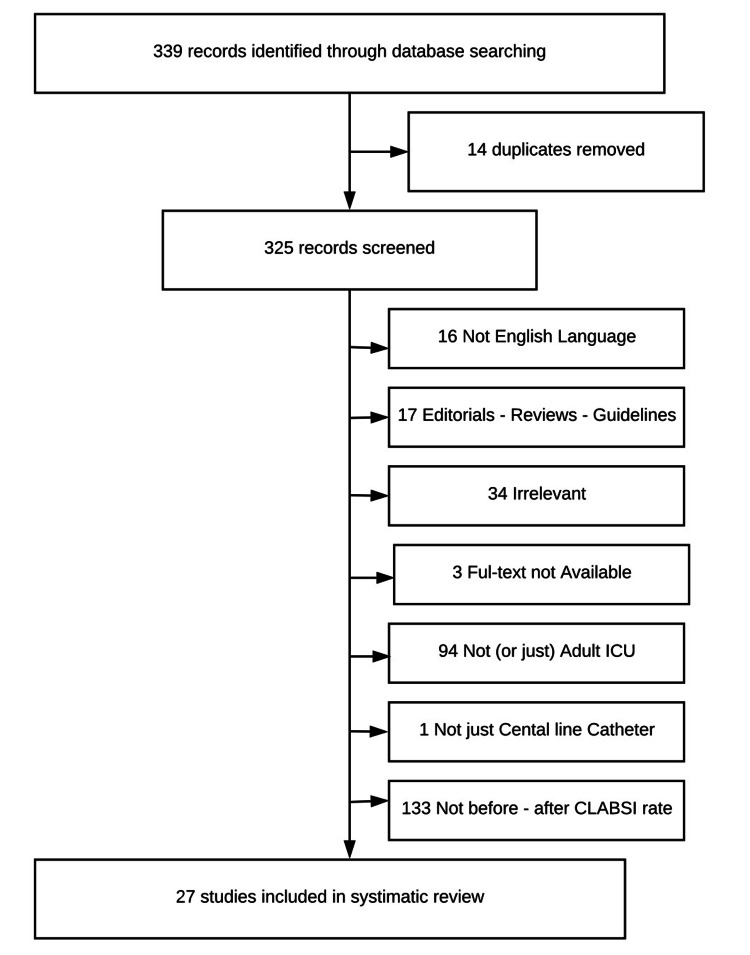
Flow diagram illustrating the inclusion of studies CLABSI: central line-associated bloodstream infection

The search algorithm yielded 339 potentially relevant articles (Medline, CINAHAL, Cochrane Database of Systematic Reviews). Among them, we found duplication of 14 studies; 16 were not in the English language, 17 were editorials, reviews, or guidelines, 34 were irrelevant to the subject, and in three articles, full-text was not available; 94 were not associated with ICU settings (n=94 studies) and 134 were not before/after CLABSI rate methodology. Therefore, they were excluded. The remaining 27 studies met the inclusion criteria and were included in the study (Figure 1) [5,19-44].

Study Characteristics

The characteristics of the studies included are summarized in Table 1. All studies were educational interventions (ie, lecture, simulation, seminar, workshop, feedback, bundle, checklist, etc.) for central lines (insertion or maintenance or both) in an adult ICU setting, with documentation of the CLABSI incidence expressed per 1,000 catheter days, pre-and post-intervention.

Ten studies were conducted in US institutions [5,19-27]; the remaining were conducted in Brazil (n=3) [28-30], Korea (n=1) [31], Saudi Arabia (n=2) [32,33], Germany (n=1) [34], India (n=2) [35,36], Turkey (n=1) [37], Canada (n=3) [38-40], Spain (n=1) [41], Bahrain (n=1) [42], and Argentina (n=1) [43]. One study was conducted in 15 countries by the INICC [44]. All studies were undertaken in adult ICUs (n=27) [5,19-44].

Two studies were associated with surgical ICUs (n=2) [25,31] and five studies with medical ICUs [26,29,35,39,42]. Five studies were undertaken in both surgical and medical ICUs [22,23,28,30,40]. One study was conducted in trauma ICU [32]. The remaining studies were undertaken in general ICUs (n=15) [5,19-21,24,27,33,34,36-38,41-44]. Three of them focused only on the physicians as the target population for the intervention [5,22,40], while the remaining 24 studies focused on both physicians' and nurses' groups (Table 2).

**Table 2:** Summary of study characteristics CLABSI: central line-associated bloodstream infection; INICC: International Nosocomial Infection Control Consortium; MINORS: Methodological Index for Non-randomized Studies

Study (first author, year, country)	Location	Intervention	Target population	Study design	Study duration (months)	Number of patients	CLABSI before	CLABSI after	P-value	MINORS score
Park et al., 2017, Korea	1 surgical ICU	Education, feedback, bundle	Nurses + physicians	Prospective intervention study	9 months	1,684 central line-days	6.9/1,000 catheter days	1.8/1,000 catheter days	0.036 (<0.001)	11
Guerin et al., 2010, USA	1 medical ICU + 1 surgical ICU	Online training IV team	Nurses + physicians	Before-after surveillance study	36 months	Pre: 4,415 catheter days, post: 2,825 catheter days	5.7/1,000 catheter days	1.1/1,000 catheter days	0.004 (<0.001)	7
Walz et al., 2015, USA	8 ICUs	Bundle, checklist, eLearning module, quality rounds	Nurses + physicians	Observational study	96 months	Not mentioned	5.86/1,000 catheter days	0.33/1,000 catheter days	<0.001	8
Mazi et al., 2014, Saudi Arabia	23-bed trauma ICU	Bundle, course, discussion	Nurses + physicians	Prospective study	24 months	13,699 patient days	3.8/1,000 catheter days	1.5/1,000 catheter days	0.43	6
Hansen et al., 2014, Germany	32 ICUs	Lectures and bundles	Nurses + physicians	Multi-center interventional study with before-after design	48 months	266,471 central-line days	2.29/1,000 catheter days	1.64/1,000 catheter days	0.001	12
Allen et al., 2014, Canada	1 medical ICU + 1 surgical ICU	Simulation training in central line insertion	Physicians	Simulation study RTS	37 months	30,553 catheter days	Medical ICU: 2.72/1,000 catheter days; surgical ICU: 1.09/1,000 catheter days	Medical ICU: 0.4/1,000 catheter days; surgical ICU: 1,14/1,000 catheter days	0.01, 0.86	13
Khalid et al., 2013, Saudi Arabia	1 ICU	Lecture and bundles	Nurses + physicians	Clinical study	24 months	11,730 catheter days	6.9/1,000 catheter days	0.35/1,000 catheter days	<0.001	12
Jaggi et al., 2013, India	16 ICUs	INICC multidimensional approach: (1) bundle of infection control interventions, (2) education, (3) outcome surveillance, (4) process surveillance, (5) feedback of CLABSI rates, and (6) performance feedback of infection control practices	Nurses + physicians	Prospective before-after cohort study	24 months	35,650 patients, 90,370 catheter days	6.4/1,000 catheter days	3.9/1,000 catheter days	0.0007	12
Leblebicioglu et al., 2013, Turkey	13 ICUs	INICC multidimensional approach: (1) bundle of infection control interventions, (2) education, (3) outcome surveillance, (4) process surveillance, (5) feedback of CLABSI rates, and (6) performance feedback of infection control practices	Nurses + physicians	Active, prospective surveillance, before-after study	88 months	4,017 patients, 3,129 catheter days	22.7/1,000 catheter days	12/1,000 catheter days	0.0007	9
Exline et al., 2013, USA	1 ICU	Bundle checklist, education, feedback	Nurses + physicians	Observational cohort study	24 months	11,271 catheter days	2.65/1,000 catheter days	1.24/1,000 catheter days	0.019	10
IIan et al., 2012, Canada	2 ICUs	Checklist and reminders	Nurses + physicians	Prospective observational study	24 months	1,010 patients, 820 catheter days	3.5/1,000 catheter days	0.0/1,000 catheter days	<0.0001	9
Burden et al., 2012, USA	24-bed ICU	Online courses simulation-based catheter-insertion course	Physicians	Pre- and post-intervention retrospective observational investigation	48 months	6,059 patients	6.47/1,000 catheter days	2.44/1,000 catheter days	<0.001	9
Render et al., 2011, USA	174 medical, cardiac, surgical, and mixed ICUs	Bundle and feedback	Nurses + physicians	Observational cohort study	36 months	104,000 patients per year, 312,000 patients on the study	3.8/1,000 catheter days	1.8/1,000 catheter days	<0.01	9
Ong et al., 2011, USA	1 trauma surgical ICU	Simulation, frequent audits, and staff motivation	Nurses + physicians	Retrospective study	114 months	8,481 patients	6.1/1,000 catheter days	0.3/1,000 catheter days	<0.001	7
Rosenthal et al., 2010, INICC Consortium, 15 countries	68 ICUs	INICC multidimensional approach: (1) bundle of infection control interventions, (2) education, (3) outcome surveillance, (4) process surveillance, (5) feedback of CLABSI rates, and (6) performance feedback of infection control practices	Nurses + physicians	Time-sequence analysis	13 months	53,719 patients, 190,905 catheter days	16.3/1,000 catheter days	10.1/1,000 catheter days	<0.001	10
Pérez Parra et al., 2017, Spain	3 ICUs	Short lecture	Nurses + physicians	Observational pre-and post-intervention study	19 months	22,243 catheter days	4.22/1,000 catheter days	2.94/1,000 catheter days	<0.03	8
Marra et al., 2010, Brazil	1 medical-surgical ICU and 2 step-down units (SDUs)	Bundles and performance monitoring	Nurses + physicians	Quasi-experimental study	48 months	99,165 patient days, 51,382 catheter days	6.4 CLABSIs/1,000 catheter days	3.2 CLABSIs/1,000 catheter days	<0.001	10
Lobo et al., 2010, Brazil	2 medical ICUs	ICU A - tailored, continuous intervention: 1. observation, 2. feedback, 3. lectures, and 4. poster; ICU B - a single lecture	Nurses + physicians	Prospective observational study	30 months	3,115 catheter days, 2,537 catheter days; total: 5,652	ICU A: 12/1,000 catheter days; ICU B: 16.2/1,000 catheter days	ICU A: 0/1,000 catheter days; ICU B: 0/1,000 catheter days; but after 6 months, 13.7/1,000 catheter days	<0.001	9
Barsuk et al., 2009, USA	1 medical ICU + 1 surgical ICU	Simulation training course	Physicians	Observational education cohort study	32 months	23,620 catheter days	3.2/1,000 catheter days	0.5/1,000 catheter days	0.001	9
Santana et al., 2008, Brazil	2 medical-surgical ICUs	Feedback and lecture	Nurses + physicians	Interventional study	9 months	186 patients, 3,152 catheter days	9.5/1,000 catheter days	5.4/1,000 catheter days	0.04	8
Galpern et al., 2008, USA	1 ICU	Bundle	Nurses + physicians	Multidisciplinary study	24 months	9,938 catheter days	5/1,000 catheter days	0.9/1,000 catheter days	<0.001	9
Alkhawaja et al., 2019, Bahrain	1 ICU	INICC multidimensional approach: (1) bundle of infection control interventions, (2) education, (3) outcome surveillance, (4) process surveillance, (5) feedback of CLABSI rates, and (6) performance feedback of infection control practices	Doctors and nurses	Prospective, before-after surveillance, cohort, observational study	36 months	2,320 patients, 13,692 catheter days	10.4/1,000 catheter days	1.20/1,000 catheter days	0.001	10
Coopersmith et al., 2004, USA	1 surgical ICU in a referral hospital	Behavioral intervention (poster and hands-on demonstration)	Nurses + physicians	Before-after trial	15 months	6,152 catheter days	3.4/1,000 catheter days	2.8/1,000 catheter days	0.04	10
Rosenthal et al., 2018, Argentina	14 ICUs	INICC multidimensional approach: (1) bundle of infection control interventions, (2) education, (3) outcome surveillance, (4) process surveillance, (5) feedback of CLABSI rates, and (6) performance feedback of infection control practices	Doctors and nurses	Prospective, pre-post surveillance study	24 months	3,940 patients, 20,777 catheter days	9.6/1,000 catheter days	4.1/1,000 catheter days	0.001	10
Warren et al., 2004, USA	19-bed medical ICU	Self-study module, observation, and feedback	Nurses + physicians	Pre-and post-intervention observational study	48 months	15,334 catheter days	9.4/1,000 catheter days	5.5/1,000 catheter days	0.019	10
Azim et al., 2019, India	1 medical ICU	Lecture and demonstration	Nurses	Quasi-experimental study	12 months	628 patients, 3,070 catheter days	12.5/1,000 catheter days	8.6/1,000 catheter days	0.02	8
Paquet et al., 2019, Canada	1 trauma-medical-surgical ICU	Lecture and audit	Nurses	Pre-and post-study	24 months	3,000 patients, 10,144 catheter days	2.36/1,000 catheter days	0/1,000 catheter days	0.0001	9

Description of Interventions

The educational interventions varied according to the study, but they all used a combination of different modalities. The most common educational tools were for bundles alone [11,24,31], or in combination with other interventions. Three studies used bundles in combination with lectures [28,33,34], five studies used the INICC multidimensional approach [36,37,42-44], three studies [5,22,40] used simulation training only for physicians in central line insertion. Other interventions used were education, bundles, and feedback checklist [19], bundles and feedback [20], bundles, courses, and discussion [32], checklist and remainder [38], bundles, checklists, eLearning modules, and quality rounds [27], online courses and checklists [5], simulation training, frequent audits, and staff motivation [21], short lectures [41], bundles and performance monitoring [28], feedback and lectures [30], lectures and bedside demonstration [35], lectures and audits [39], observation, feedback, lectures, and posters [29], behavioral interventions (posters and hands-on demonstration) [25], and self-study module, observation, and feedback [26]. The duration of each intervention was also highly variable, ranging from one-day lecture [41] to sustained interventions lasting up to nine months [30] and even years [21]. Bundles used in the studies were associated with CVC insertion and maintenance.

Efficacy of Interventions

All studies included in this review reported CLABSI rates before and after an educational intervention. Only one study included in this review did not find a statistically significant reduction in CLABSI rates after the implementation of the intervention [34]. All the other included studies found evidence for the substantial efficacy of the educational intervention.

Durability of Intervention Effect

The sustainability of the intervention effect seems to be associated with longer study duration and multidimensional approaches. In the study by Walz et al. [27], interventions began in 2004 and lasted until 2011. The intervention includes bundles, checklists, an eLearning module, and quality rounds. The initial CLABSI rate was 5.86 per 1,000 catheter days (2004), which was reduced to 0.33 by 2012 (p<0.0001).

A CLABSI rate reduction was also observed in Ong et al.'s [21] interventions, which began in 2001 and lasted until 2009. It included standardization of line insertion and maintenance processes, the development of a mandatory education program incorporating line insertion simulation practice sessions, frequent audits, and ICU staffing modifications. The CLABSI rate was 6.1 per 1,000 catheter days (2004), which was reduced to 0.3 per 1,000 catheter days by 2012 (p<0.0001). 

Assessment of Methodologic Quality of Included Studies

The study rationale was easily identifiable in all studies [5,19-44]. In all of them, the study design was appropriate for the study question, and the majority described the design in sufficient detail. However, two studies [29,40] chose a similar comparison group for their study. None of the studies was a cluster randomized controlled trial.

The setting in all of the studies under which the interventions were carried out was well described. However, a detailed description of the follow-up period appropriate to the aim of the study was found in only two studies [29,31]. The methods used for statistical testing were described in all studies (Table 2).

Discussion

Our systematic review shows that a variety of educational strategies have been studied for the prevention of CLABSI, which targeted nurses and physician groups. We found that the systematic application of educational interventions can decrease rates of CLABSI; however, it is difficult to determine the most effective educational intervention due to the presence of a variety of approaches.

Studies from developing countries [36,37,43,44] that implemented the INICC multidimensional approach showed a statistically significant reduction in the CLABSI rates. Educational interventions that were undertaken in developing countries found substantial benefits similar to the studies that were undertaken in developed countries. Although the educational strategies were highly variable and multidimensional, most of the interventions emphasized the need for the adoption of insertion and maintenance bundles for CLABSI prevention.

In all of the studies included in the current review, nurses were the main target population except in three [5,22,40]. This probably shows the importance given to the nurses' role in the insertion and maintenance of CVCs. Nurses are providing continuing care to ICU patients and have a key role in the quality of care provided [45].

The simplest intervention found was a short lecture of 15 minutes among the 10 main points of Infectious Diseases Society of America/Centers for Disease Control and Prevention (IDSA-CDC) guidelines for the prevention of intravascular catheter-related infections, which surprisingly led to a statistically significant decrease in CLABSI rates (p<0.03) [41].

The lack of a detailed description of the content of the educational interventions, including an assessment of validation of the intervention, hindered the generalizability of findings. Issues such as the educational background, years of experience, and hours of clinical training of the staff may also affect the type and effectiveness of the intervention. Whether these interventions are sustainable in time or should be periodically repeated are also issues that should be addressed in future studies, especially taking into consideration two studies in this review [29,31], since their conclusions showed that low CLABSI rates can only be sustained with repeated and continued infection prevention education.

Limitations

The current study has certain limitations. We included studies in the English language only, and hence data from studies in other languages may be missing. The studies were very heterogeneous and we were unable to determine the most effective type of education intervention for CLABSI rates. We only analyzed studies pertaining to ICU settings, and hence our findings cannot be generalized to other settings. Moreover, we only included studies that assessed CLABSI rates and rejected other definitions for bloodstream infections associated with CVC.

## Conclusions

This systematic review identified several educational interventions capable of reducing CLABSI rates, either in combination or alone. However, to maintain reduced CLABSI rates post-intervention, regular follow-ups, resource support, and multifaceted cooperative approaches may be essential.
